# Burden of early-onset colorectal cancer along with attributable risk factors from 1990 to 2019: a comparative study between China and other G20 countries

**DOI:** 10.1186/s12889-023-16407-y

**Published:** 2023-07-31

**Authors:** Quanhui Li, Miao Yu, Haiguang Lv, Le Zhang, Yang Deng, Hualong Yu

**Affiliations:** 1 Department of General Surgery, The Second Hospital, Cheeloo College of Medicine, Shandong University, No.247 Beiyuan Road, Jinan, Shandong Province 250000 China; 2 Department of Colorectal and Anal Surgery, The Second Hospital, Cheeloo College of Medicine, Shandong University, No.247 Beiyuan Road, Jinan, Shandong Province 250000 China; 3 Department of Clinical Laboratory, Second Affiliated Hospital of Shandong First Medical University, No.706 Taishan Road, Tai’an, Shandong Province 271000 China; 4 School of Public Health, Shandong First Medical University & Shandong Academy of Medical Sciences, No.6699 Qingdao Road, Jinan, Shandong Province 250000 China

**Keywords:** Early-onset colorectal cancer (EOCRC), Incidence, Mortality, Disability-adjusted life years (DALYs), Risk factor, Temporal trends

## Abstract

**Purpose:**

The credible data about the burden of early-onset colorectal cancer (EOCRC) in China when compared to other countries in the group of twenty (G20) remained unavailable. We aimed to assess the burden and trends of EOCRC and attributable risk factors in China. Meanwhile, the comparison in the burden and attributable risk factors between China and other G20 countries was also evaluated.

**Methods:**

Data on the incidence, prevalence, mortality, disability-adjusted life years (DALYs), and attributable risk factors of EOCRC in China were obtained from Global Burden of Diseases, Injuries, and Risk Factors Study (GBD) 2019 and compared with other G20countries. Temporal trends of age-standardized rates for incidence, prevalence, mortality, and DALYs were evaluated by estimated annual percentage change (EAPC). The autoregressive integrated moving average (ARIMA) model was used to forecast the incidence, mortality, and DALY rates of EOCRC in China from 2020 to 2029.

**Results:**

From 1990 to 2019, the age-standardized incidence rate (ASIR) and age-standardized prevalence rate (ASPR) of EOCRC in China increased with the EAPCs of 4.61 [95% confidence interval (*CI*): 4.45–4.77] and 5.82 (95% *CI*: 5.60–6.05). When compared to G20 countries, China was ranked 13^th^ in the ASIR in 1990 and then increased to 2^nd^ in 2019, second only to Japan. The ASPRs increased in all G20 countries, being highest in Saudi Arabia, followed by China and Mexico. Moreover, China had the highest age-standardized mortality rate and highest age-standardized DALY rate in 2019. In China, the five leading risk factors, for both sexes, were diet low in milk [18.54% (95% UI: 12.71–24.07)], diet low in calcium [15.06% (95% UI: 10.70–20.03)], alcohol use [12.16% (95% UI: 8.87–15.64)], smoking [9.08% (95% UI: 3.39–14.11)], and diet high in red meat [9.08% (95% UI: 3.39–14.11)] in 2019. Over the next 10 years, ASIR, ASMR, and age-standardized DALY rate of EOCRC will increase continuously in males and females.

**Conclusion:**

The burden of EOCRC in China and other G20 countries is worrisome, indicating that coordinated efforts are needed to conduct high-quality researches, allocate medical resources, adjust screening guidelines, and develop effective treatment and prevention strategies in the G20 countries.

**Supplementary Information:**

The online version contains supplementary material available at 10.1186/s12889-023-16407-y.

## Introduction

Globally, colorectal cancer (CRC) is one of the major reasons for the global burden of cancer, with more than 1.9 million new cases and 935,000 deaths in 2020 [1]. Overall, CRC ranks as the third most frequently diagnosed cancer and the second leading causes of cancer-associated deaths [1, 2]. In the past three decades, the age-standardized incidence and mortality rates of CRC either remained steady or declined in high Socio-demographic Index (SDI) countries such as the United States, Australia, and Germany [3]. However, large increases in the CRC incidence rates occurred in low and middle SDI countries [4]. It is noted that China has become the country with the largest number of new cases and deaths from CRC annually [5, 6]. The incidence rate of early-onset CRC (EOCRC) is increasing worldwide, which is defined as that diagnosed in individuals aged younger than 50 years. Up to 90% of all patients with newly diagnosed CRC are aged 50 years and over, namely, late-onset CRC (LOCRC), but EOCRC accounts for 10%-12% of CRC cases [7, 8]. A previous study revealed an increasing trend in the incidence of CRC in China from 1990 to 2016, with the most significant increase in the cases under the age of 50 [9]. Patients with EOCRC are prone to possess an underappreciation of symptoms, lack of awareness about CRC and early screening, have more reluctance to seek medical assistance, leading to delayed diagnosis and advanced stage at diagnosis [10]. In addition, EOCRC patients experienced significantly longer time to diagnosis and longer duration of symptoms compared to patients with LOCRC [10, 11]. Thus, EOCRC causes a large burden of disease among young adults.

It is reported that 70–75% of CRC cases are associated with modifiable risk factors such as smoking, alcohol use, high body-mass index, diet high in processed and red meat, whereas the remaining 25–30% of cases are related to non-modifiable risk factors including genetic factors, personal history of polyps or adenoma, or family history of CRC [4, 12]. The Global Burden of Diseases, Injuries, and Risk Factors Study (GBD) 2019 has verified that 58.2% of cancer related-deaths and 57.6% of disability-adjusted life years (DALYs) due to CRC were approximately attributable to well recognized risk factors [13]. The reasons for the rapid increasing trends of EOCRC incidence and mortality are unclear, but they are likely to be affected a combination of modifiable and non-modifiable risk factors, such as excess body weight, lacking of physical activity, diabetes mellitus, male, black or Asian, family history of CRC, and personal history of inflammatory bowel disease [14–16]. However, whether known risk factors play similarly critical roles in EOCRC incidence is poorly understood. Also, identifying risk factors is essential to optimize guidelines for prevention and early detection for EOCRC.

The Group of 20 (G20), which is widely recognized as one of the largest international economic cooperation fora, accounts for almost two-thirds of the world’s population [17]. The burden of diseases among G20 countries are inextricably related to their economic environment and medical condition [18]. Previous studies have identified the burden of EOCRC varied across geographic locations and socioeconomic statuses [19, 20]. However, a comprehensive analysis regarding disease burden and long-term trends along with attributable risk factors of EOCRC in China and other member countries of G20 has not been available. A better understanding of the current status and future trends of disease burden can promote effective prevention and aid policy decision-makers to allocate health care resources precisely and efficiently. Therefore, in this study, we utilized data from the GBD 2019 to quantify the trends in incidence, prevalence, mortality, DALYs of EOCRC in China and compared these indicators to other G20 countries from 1990 to 2019, and the contributions of risk factors. We also predicted the burden up to 2029 in China.

## Methods

### Study design and data sources

This study was designed as a secondary analysis of publicly available anonymized aggregate data from the GBD 2019, which comprehensively assessed the burden of 369 diseases and injuries, and 87 behavioral, environmental and occupational, and metabolic risk factors in 204 countries and territories from 1990 to 2019. The GBD 2019 was considered as a standardized framework for integrating, validating, analyzing, and disseminating the disease burden and for evaluating the burden of premature death, health loss, and disability due to disease, injury, and risk factors in diverse populations [21]. Data assembled in the GBD 2019 were derived from multiple sources, including the published literature reviews, cohort studies, cross-sectional studies, case reports, Global Health Databases, vital registration databases, sample registration systems, household surveys, censuses and health and demographic surveillance websites [21, 22]. The DisMod-MR 2.1, a Bayesian meta-regression tool, was used to pool the incidence and mortality data and generate location-year-age-sex-specific estimates in GBD 2019 [21]. More details about the methodology of GBD 2019 have been previously described [21, 22]. Herein, we obtained and analyzed the data on incidence, prevalence, mortality, DALYs, and related risk factors of EOCRC in China during 1990–2019, stratified by sex. In addition, we compared EOCRC estimates with other member countries of the G20 (an international forum for the governments from 20 major economies including Argentina, Australia, Brazil, Canada, European Union, France, Germany, India, Indonesia, Italy, Japan, Mexico, Republic of Korea, Russian Federation, Saudi Arabia, South Africa, Turkey, United Kingdom, and United States of America), and the European Union is considered as a whole in this analysis. DALYs are regarded as summary indicators to measure the disease burden due to disability and premature death in the GBD study [21]. DALYs are calculated as the sum of the YLLs and YLDs, and one DALY is comparable to one lost year of healthy life.The Global Health Data Exchange (GHDx) query tool (http://ghdx.healthdata.org/) was used to collect the data analyzed in this study. Our study followed the Guidelines for Accurate and Transparent Health Estimates Reporting (GATHER) to ensure the transparency, reliability and replicability [23].

### Definition of EOCRC

We regarded all cases coded C18-C21, D01.0-D01.2, and D12-D12.8 as CRC according to the 10th revision of International Classification of Diseases [4]. EOCRC were defined as CRC cases diagnosed at an age of 50 years or younger.

### Risk factors

Disease burden attributable to each risk factor was estimated according to the comparative risk assessment framework in the GBD 2019. Ten risk factors that have a non-zero contribution to deaths and DALYs of CRC were selected, including five dietary factors (diet high in processed meat, diet high in red meat, diet low in calcium, diet low in fiber, and diet low in milk), three behavioral factors (alcohol use, smoking, and low physical activity), and two metabolic factors (high body-mass index and high fasting plasma glucose) [4]. Detailed definitions of these risk factors and methods for quantifying the proportions of the burden of EOCRC attributable to these risk factors have been described previously [22]. The percentage contribution of these ten risk factors to the DALYs of EOCRC in 2019 were assessed in this study.

### Statistical analysis

The quantity of incidence, prevalence, mortality, and DALYs mainly covers the number, percent, and rate. The 95% uncertainty intervals (UIs) were demonstrated for each estimated quantity. Each estimate such as incident cases and age-standardized incidence rate in GBD study was calculated 1,000 times, with each time sampling from distributions for data inputs, data transformations, and model choice. The 95% UIs were determined by the 25th and 975th values of the 1,000 values after ordering them from smallest to largest [21]. Age-standardized rates for incidence, prevalence, mortality, and DALYs were computed by employing direct standardization to the World Health Organization (WHO) world standard population age-structure from 2000 to 2025 [24]. We calculated the estimated annual percentage change (EAPC) in the age-standardized rates for incidence, prevalence, mortality, and DALYs to assess the temporal trends in the burden of EOCRC during 1990–2019. EAPC was a widely accepted measure to quantificationally describe the trend of age-standardized rates over specific time intervals, and it was calculated based on the regression model fitted to the natural logarithm of the rates. The regression model was defined as: *ln* (rate) = *α* + *β* × (calendar year) + *ε*, and EAPC was calculated as 100 × (exp(β)-1). The 95% confidence interval (*CI*) was also determined by the linear regression model. When EAPC and the lower limit of 95% *CI* are positive, that age-standardized rate is considered to be in an increasing trend. Conversely, if EAPC and the upper limit of 95% *CI* are less than 0, the age-standardized rate shows a descending trend. The autoregressive integrated moving average (ARIMA) (*p*, *d*, *q*) model was selected to forecast the incidence, mortality, and DALY rates of EOCRC from 2020 to 2029. The letters *p*, *d*, *q* represented the orders of autoregression, degree of difference, and order of moving average, respectively. More details about ARIMA model were provided elsewhere [25, 26]. We constructed this model in the following steps: stationary test, model identification, parameter estimation, model diagnosis, and model prediction. The Augmented Dickey–Fuller (ADF) test is used to determine whether the series is stationary or not. If the rates were not stationary series, logarithmic transformation was performed to transform them into stationary series. The autocorrelation function (ACF) and the partial autocorrelation function (PACF) were used to determine the appropriate model parameters (*p* and *q*). The optimal ARIMA (*p*, *d*, *q*) model was evaluated by the Akaike information criterion (AIC) and the Bayesian information criterion (BIC). We used the Ljung–Box Q test, ACF, and PACF of residuals to judge whether the residuals of the optimal model meet the requirements of white noise sequences. Then, the model was constructed and used to forecast the rates. All statistical analyses and graphics were performed by software package R version 4.1.1. *P* < 0.05 was considered statistically significant.

## Results

### Disease burden of EOCRC in China

In China, there were 23,971 (95% UI: 20,744–27,552) incident cases of EOCRC in 1990 and 87,383 (95% UI: 72,880–103,288) cases in 2019, representing an increase of 264.54% from 1990 to 2019. Meanwhile, the global incident cases of EOCRC were 225,736 (95% UI: 207,658–246,756) in 2019, increasing from 94,707 (95% UI: 90,421–99,416) in 1990. The age-standardized incidence rates (ASIRs) of EOCRC in China were 3.59 (95% UI: 3.10–4.12) in 1990 and 12.12 (95% UI: 10.11–14.33) in 2019 per 100,000 population, which were higher than the global average of 3.49 (95% UI: 3.33–3.67) in 1990 and 5.74 (95% UI: 5.28–6.27) in 2019 per 100,000 population. When compared to G20 countries, China was ranked 13^th^ in the ASIR in 1990 and then increased to 2^nd^ in 2019, second only to Japan (Table 1). EOCRC accounted for 121,016 (95% UI: 105,534–138,358) prevalent cases in 1990 and 590,804 (95% UI: 494,021–697,827) cases in 2019 in China, comprising 23.08% of global prevalent cases in 1990 and 41.57% in 2019. Age-standardized prevalence rate (ASPR) of EOCRC in China jumped from the 13^th^ in 1990 to the 3^rd^ in 2019 (Table 2). It is worthwhile to point out China had the highest number of deaths caused by EOCRC in 1990 [14,851 (95% UI: 12,694–17,187)] and 2019 [26,274 (95% UI: 21,892–31,077)] among the G20 countries. Moreover, China had the highest age-standardized mortality rate (ASMR) [3.65 (95% UI: 3.04–4.31)] per 100,000 population in 2019 (Table 3). Worldwide, approximately 2.52 (95% UI: 2.37–2.66) million and 4.26 (95% UI: 3.94–4.59) million DALYs due to EOCRC were reported in 1990 and 2019, with more than 30% of global DALYs coming from China. Japan was the country with the highest age-standardized DALY rate [166.53 (95% UI: 162.97–169.95)] per 100,000 population in 1990, and China had the highest age-standardized DALY rate [181.04 (95% UI: 152.57–211.83)] per 100,000 population in 2019 (Table 4).

**Table 1:** Incidence of early-onset colorectal cancer in G20 countries and global average, and temporal trends from 1990 to 2019

**Location**	**Cases (95% UI)**			**Age-standardized incidence rate, per 100 000 (95% UI)**		**1990–2019** **EAPC (95% *CI*)**
	**1990**	**2019**	**1990–2019** **Change (%)**	**1990**	**2019**	
China	23,971 (20,744 to 27,552)	87,383 (72,880 to 103,288)	264.54 (186.71 to 359.15)	3.59 (3.10 to 4.12)	12.12 (10.11 to 14.33)	4.61 (4.45 to 4.77)
Global	94,707 (90,421 to 99,416)	225,736 (207,658 to 246,756)	138.35 (116.38 to 162.62)	3.49 (3.33 to 3.67)	5.74 (5.28 to 6.27)	1.73 (1.65 to 1.80)
Argentina	689 (645 to 735)	1632 (1226 to 2130)	136.87 (76.17 to 210.77)	4.33 (4.06 to 4.62)	7.04 (5.29 to 9.18)	1.37 (1.22 to 1.51)
Australia	767 (721 to 813)	1306 (981 to 1710)	70.37 (27.04 to 123.16)	8.53 (8.03 to 9.05)	11.32 (8.50 to 14.82)	0.92 (0.78 to 1.06)
Brazil	1890 (1831 to 1957)	5615 (5286 to 5915)	197.10 (177.76 to 217.28)	2.47 (2.39 to 2.55)	4.86 (4.58 to 5.12)	2.40 (2.15 to 2.65)
Canada	1165 (1085 to 1249)	1761 (1309 to 2319)	51.19 (11.23 to 103.40)	7.90 (7.36 to 8.47)	10.89 (8.09 to 14.34)	1.23 (1.00 to 1.48)
European Union	16,499 (16,179 to 16,837)	20,515 (17,788 to 23,500)	24.34 (7.30 to 41.96)	6.89 (6.75 to 7.03)	9.05 (7.85 to 10.37)	0.80 (0.57 to 1.04)
France	1720 (1607 to 1837)	2261 (1644 to 3053)	31.45 (-5.13 to 76.66)	5.90 (5.51 to 6.30)	7.95 (5.78 to 10.74)	0.74 (0.47 to 1.00)
Germany	2947 (2762 to 3149)	3036 (2283 to 4097)	3.02 (-22.87 to 40.20)	7.39 (6.93 to 7.90)	8.46 (6.36 to 11.41)	0.64 (-0.14 to 1.43)
India	5085 (4458 to 5773)	14,736 (12,218 to 17,329)	189.79 (127.50 to 253.76)	1.21 (1.06 to 1.37)	1.94 (1.61 to 2.28)	1.48 (1.36 to 1.61)
Indonesia	2216 (1614 to 2733)	6765 (4855 to 8818)	205.24 (131.28 to 308.35)	2.34 (1.71 to 2.89)	4.74 (3.40 to 6.18)	2.45 (2.27 to 2.63)
Italy	2256 (2132 to 2384)	2646 (2110 to 3240)	17.29 (-15.86 to 45.34)	7.85 (7.42 to 8.30)	10.44 (8.32 to 12.78)	0.92 (0.71 to 1.13)
Japan	6816 (6597 to 7029)	6650 (5542 to 7920)	-2.45 (-19.00 to 15.63)	10.49 (10.15 to 10.82)	12.85 (10.71 to 15.3)	0.48 (0.29 to 0.67)
Mexico	676 (660 to 691)	2943 (2500 to 3433)	335.64 (268.43 to 408.59)	1.59 (1.55 to 1.63)	4.44 (3.77 to 5.18)	3.68 (3.55 to 3.82)
Republic of Korea	1001 (923 to 1080)	2408 (1929 to 2950)	140.66 (91.68 to 199.68)	3.87 (3.57 to 4.17)	9.25 (7.4 to 11.33)	2.70 (2.17 to 3.23)
Russian Federation	3908 (3562 to 4156)	6551 (5526 to 7685)	67.63 (43.87 to 94.97)	5.26 (4.80 to 5.60)	9.57 (8.07 to 11.22)	1.59 (1.18 to 2.01)
Saudi Arabia	94 (63 to 136)	1147 (810 to 1598)	1120.89 (607.44 to 1923.12)	1.16 (0.78 to 1.68)	4.71 (3.33 to 6.56)	5.14 (4.94 to 5.34)
South Africa	472 (417 to 535)	789 (675 to 913)	67.20 (35.53 to 104.98)	2.48 (2.19 to 2.81)	2.59 (2.22 to 3.00)	-0.19 (-0.37 to -0.01)
Turkey	1057 (790 to 1366)	2429 (1888 to 3120)	129.69 (56.42 to 229.57)	3.53 (2.64 to 4.56)	5.32 (4.14 to 6.83)	1.18 (0.75 to 1.62)
United Kingdom	1964 (1917 to 2018)	2707 (2215 to 3273)	37.86 (12.13 to 66.96)	6.90 (6.73 to 7.09)	8.88 (7.27 to 10.74)	0.97 (0.85 to 1.10)
United States of America	10,497 (10,214 to 10,761)	16,735 (14,188 to 19,774)	59.43 (34.93 to 89.58)	7.84 (7.63 to 8.04)	11.12 (9.42 to 13.13)	1.21 (0.97 to 1.46)

*G20* Group of 20, *UI* Uncertainty interval, *EAPC* Estimated annual percentage change, *CI* Confidence interval

**Table 2:** Prevalence of early-onset colorectal cancer in G20 countries and global average, and temporal trends from 1990 to 2019

**Location**	**Cases (95% UI)**			**Age-standardized prevalence rate, per 100 000 (95% UI)**		**1990–2019** **EAPC (95% CI)**
	**1990**	**2019**	**1990–2019** **Change (%)**	**1990**	**2019**	
China	121,016 (105,534 to 138,358)	590,804 (494,021 to 697,827)	388.20 (288.25 to 513.29)	18.10 (15.79 to 20.7)	81.97 (68.55 to 96.82)	5.82 (5.60 to 6.05)
Global	524,279 (502,878 to 548,841)	1,421,369 (1,308,279 to 1,555,222)	171.11 (147.12 to 198.24)	19.33 (18.54 to 20.24)	36.12 (33.25 to 39.52)	2.24 (2.14 to 2.33)
Argentina	3450 (3217 to 3700)	9442 (7107 to 12,301)	173.67 (106.33 to 258.25)	21.70 (20.23 to 23.27)	40.71 (30.65 to 53.04)	1.85 (1.69 to 2.01)
Australia	5187 (4854 to 5533)	9612 (7312 to 12,478)	85.30 (36.93 to 141.97)	57.72 (54.02 to 61.57)	83.30 (63.37 to 108.15)	1.27 (1.11 to 1.42)
Brazil	8898 (8593 to 9229)	31,259 (29,384 to 33,028)	251.30 (228.26 to 275.14)	11.61 (11.21 to 12.04)	27.07 (25.44 to 28.60)	2.94 (2.68 to 3.20)
Canada	8224 (7609 to 8888)	13,106 (9810 to 17,261)	59.36 (18.31 to 112.48)	55.77 (51.60 to 60.27)	81.05 (60.66 to 106.74)	1.45 (1.21 to 1.68)
European Union	103,078 (100,396 to 106,116)	143,843 (125,438 to 164,248)	39.55 (20.99 to 59.64)	43.01 (41.90 to 44.28)	63.48 (55.36 to 72.49)	1.26 (1.01 to 1.50)
France	10,916 (10,119 to 11,796)	16,166 (11,957 to 21,521)	48.10 (8.30 to 97.50)	37.42 (34.69 to 40.44)	56.86 (42.05 to 75.69)	1.27 (0.98 to 1.57)
Germany	18,962 (17,656 to 20,482)	21,808 (16,596 to 29,004)	15.01 (-12.92 to 56.25)	47.54 (44.27 to 51.35)	60.76 (46.24 to 80.80)	1.05 (0.28 to 1.82)
India	20,444 (18,038 to 23,048)	66,819 (55,780 to 77,980)	226.83 (159.31 to 296.92)	4.87 (4.29 to 5.49)	8.80 (7.34 to 10.27)	1.91 (1.80 to 2.02)
Indonesia	9605 (7176 to 11,701)	32,922 (23,962 to 42,477)	242.76 (163.67 to 350.78)	10.16 (7.59 to 12.38)	23.06 (16.78 to 29.75)	2.84 (2.70 to 2.98)
Italy	15,217 (14,208 to 16,277)	19,475 (15,610 to 23,740)	27.99 (2.26 to 59.14)	52.98 (49.47 to 56.67)	76.80 (61.56 to 93.62)	1.27 (1.05 to 1.49)
Japan	46,487 (44,584 to 48,371)	49,343 (41,199 to 58,459)	6.14 (-11.53 to 25.85)	71.56 (68.63 to 74.46)	95.34 (79.60 to 112.95)	0.83 (0.68 to 0.98)
Mexico	3390 (3292 to 3493)	17,162 (14,665 to 19,861)	406.31 (329.82 to 490.96)	7.97 (7.74 to 8.22)	25.89 (22.12 to 29.96)	4.04 (3.91 to 4.17)
Republic of Korea	5648 (5155 to 6141)	17,436 (14,106 to 21,234)	208.71 (145.46 to 282.88)	21.82 (19.92 to 23.73)	66.94 (54.16 to 81.53)	3.51 (2.91 to 4.11)
Russian Federation	22,557 (20,630 to 23,949)	42,422 (36,102 to 49,711)	88.06 (61.63 to 119.10)	30.38 (27.78 to 32.25)	61.95 (52.73 to 72.60)	2.18 (1.90 to 2.46)
Saudi Arabia	454 (317 to 647)	6893 (4891 to 9671)	1416.89 (803.60 to 2342.97)	5.62 (3.92 to 8.01)	28.30 (20.08 to 39.71)	5.90 (5.67 to 6.14)
South Africa	2031 (1811 to 2293)	3746 (3234 to 4310)	84.37 (50.32 to 125.37)	10.67 (9.51 to 12.04)	12.30 (10.62 to 14.15)	0.17 (0.03 to 0.31)
Turkey	4656 (3515 to 6034)	14,728 (11,379 to 18,964)	216.28 (116.38 to 350.68)	15.55 (11.74 to 20.15)	32.25 (24.92 to 41.53)	2.48 (2.13 to 2.83)
United Kingdom	12,584 (12,193 to 13,035)	19,210 (15,757 to 23,107)	52.66 (25.65 to 84.17)	44.19 (42.81 to 45.77)	63.02 (51.69 to 75.81)	1.37 (1.25 to 1.49)
United States of America	73,170 (70,894 to 75,636)	120,487 (103,041 to 141,173)	64.67 (40.34 to 93.95)	54.66 (52.96 to 56.51)	80.03 (68.44 to 93.77)	1.28 (1.05 to 1.51)

*G20* Group of 20, *UI* Uncertainty interval, *EAPC* Estimated annual percentage change, *CI* Confidence interval

**Table 3:** Mortality of early-onset colorectal cancer in G20 countries and global average, and temporal trends from 1990 to 2019

**Location**	**Cases (95% UI)**			**Age-standardized mortality rate, per 100 000 (95% UI)**		**1990–2019** **EAPC (95% CI)**
	**1990**	**2019**	**1990–2019** **Change (%)**	**1990**	**2019**	
China	14,851 (12,694 to 17,187)	26,274 (21,892 to 31,077)	76.91 (39.57 to 121.64)	2.22 (1.90 to 2.57)	3.65 (3.04 to 4.31)	1.80 (1.68 to 1.93)
Global	50,437 (47,475 to 53,368)	86,546 (80,162 to 93,431)	71.59 (55.24 to 87.55)	1.86 (1.75 to 1.97)	2.20 (2.04 to 2.37)	0.48 (0.40 to 0.55)
Argentina	432 (406 to 460)	782 (707 to 866)	80.77 (59.91 to 103.71)	2.72 (2.55 to 2.89)	3.37 (3.05 to 3.73)	0.50 (0.40 to 0.60)
Australia	257 (244 to 269)	282 (254 to 315)	9.96 (-1.96 to 24.11)	2.86 (2.72 to 3.00)	2.45 (2.20 to 2.73)	-0.65 (-0.80 to -0.50)
Brazil	1221 (1184 to 1266)	2785 (2630 to 2928)	128.01 (113.04 to 142.82)	1.59 (1.55 to 1.65)	2.41 (2.28 to 2.54)	1.47 (1.27 to 1.67)
Canada	342 (326 to 358)	363 (323 to 406)	6.05 (-6.41 to 20.34)	2.32 (2.21 to 2.43)	2.24 (2.00 to 2.51)	-0.06 (-0.22 to 0.09)
European Union	6834 (6723 to 6945)	5689 (5374 to 6038)	-16.75 (-21.46 to -11.85)	2.85 (2.81 to 2.90)	2.51 (2.37 to 2.66)	-0.68 (-0.84 to -0.51)
France	695 (661 to 731)	584 (511 to 658)	-16.02 (-27.56 to -4.32)	2.38 (2.27 to 2.50)	2.05 (1.80 to 2.31)	-0.94 (-1.13 to -0.75)
Germany	1145 (1088 to 1209)	791 (709 to 884)	-30.94 (-38.88 to -22.21)	2.87 (2.73 to 3.03)	2.20 (1.97 to 2.46)	-0.65 (-1.38 to 0.09)
India	3990 (3508 to 4522)	9962 (8306 to 11,872)	149.66 (97.51 to 203.83)	0.95 (0.84 to 1.08)	1.31 (1.09 to 1.56)	0.96 (0.84 to 1.08)
Indonesia	1618 (1184 to 1978)	4190 (2996 to 5533)	159.06 (94.02 to 247.58)	1.71 (1.25 to 2.09)	2.93 (2.10 to 3.88)	1.90 (1.70 to 2.11)
Italy	765 (743 to 785)	595 (558 to 629)	-22.21 (-26.96 to -17.45)	2.66 (2.59 to 2.73)	2.35 (2.20 to 2.48)	-0.54 (-0.61 to -0.47)
Japan	2248 (2208 to 2286)	1427 (1342 to 1480)	-36.51 (-39.72 to -34.04)	3.46 (3.40 to 3.52)	2.76 (2.59 to 2.86)	-1.02 (-1.25 to -0.80)
Mexico	410 (400 to 418)	1335 (1131 to 1554)	225.90 (176.30 to 281.87)	0.96 (0.94 to 0.98)	2.01 (1.71 to 2.34)	2.77 (2.64 to 2.89)
Republic of Korea	531 (489 to 569)	568 (498 to 646)	7.01 (-7.88 to 25.44)	2.05 (1.89 to 2.20)	2.18 (1.91 to 2.48)	-0.20 (-0.55 to 0.15)
Russian Federation	1866 (1701 to 1982)	2240 (1883 to 2620)	20.05 (2.44 to 39.71)	2.51 (2.29 to 2.67)	3.27 (2.75 to 3.83)	0.04 (-0.49 to 0.58)
Saudi Arabia	76 (49 to 114)	495 (357 to 686)	551.19 (270.91 to 1039.1)	0.94 (0.61 to 1.41)	2.03 (1.47 to 2.82)	2.58 (2.53 to 2.63)
South Africa	338 (301 to 381)	510 (440 to 591)	50.82 (23.03 to 86.75)	1.78 (1.58 to 2.00)	1.67 (1.44 to 1.94)	-0.52 (-0.74 to -0.31)
Turkey	737 (554 to 948)	1022 (800 to 1298)	38.71 (-3.96 to 99.64)	2.46 (1.85 to 3.17)	2.24 (1.75 to 2.84)	-0.75 (-1.17 to -0.33)
United Kingdom	781 (766 to 799)	708 (687 to 729)	-9.45 (-12.78 to -6.34)	2.74 (2.69 to 2.81)	2.32 (2.25 to 2.39)	-0.62 (-0.78 to -0.46)
United States of America	3261 (3178 to 3341)	4181 (4040 to 4335)	28.20 (22.54 to 35.55)	2.44 (2.37 to 2.50)	2.78 (2.68 to 2.88)	0.49 (0.33 to 0.65)

*G20* Group of 20, *UI* Uncertainty interval, *EAPC* Estimated annual percentage change, *CI* Confidence interval

**Table 4:** Disability-adjusted life years of early-onset colorectal cancer in G20 countries and global average, and temporal trends from 1990 to 2019

**Location**	**DALYs (95% UI)**			**Age-standardized DALY rate, per 100 000 (95% UI)**		**1990–2019** **EAPC (95% CI)**
	**1990**	**2019**	**1990–2019** **Change (%)**	**1990**	**2019**	
China	760,715 (651,767 to 876,402)	1,304,828 (1,099,585 to 1,526,715)	71.53 (37.27 to 114.41)	113.80 (97.50 to 131.11)	181.04 (152.57 to 211.83)	1.64 (1.54 to 1.75)
Global	2,516,721 (2,368,906 to 2,663,625)	4,259,922 (3,942,850 to 4,590,979)	69.26 (53.45 to 84.80)	92.79 (87.34 to 98.21)	108.25 (100.20 to 116.67)	0.42 (0.35 to 0.49)
Argentina	20,910 (19,682 to 22,238)	38,318 (34,749 to 42,352)	83.25 (62.90 to 105.45)	131.50 (123.77 to 139.85)	165.23 (149.84 to 182.62)	0.57 (0.47 to 0.66)
Australia	12,438 (11,828 to 13,053)	14,080 (12,594 to 15,714)	13.20 (0.74 to 26.96)	138.40 (131.61 to 145.25)	122.02 (109.15 to 136.19)	-0.52 (-0.66 to -0.38)
Brazil	61,450 (59,593 to 63,598)	135,881 (128,375 to 142,679)	121.12 (106.83 to 134.89)	80.17 (77.75 to 82.98)	117.66 (111.16 to 123.54)	1.37 (1.19 to 1.55)
Canada	16,704 (15,908 to 17,541)	18,010 (16,063 to 20,179)	7.82 (-4.92 to 22.42)	113.27 (107.87 to 118.95)	111.37 (99.34 to 124.78)	0.01 (-0.14 to 0.14)
European Union	329,018 (323,030 to 334,876)	274,666 (258,613 to 291,619)	-16.52 (-21.41 to -11.44)	137.30 (134.80 to 139.74)	121.22 (114.14 to 128.71)	-0.66 (-0.82 to -0.50)
France	33,675 (32,023 to 35,431)	28,271 (24,834 to 31,889)	-16.05 (-27.12 to -4.52)	115.44 (109.77 to 121.45)	99.43 (87.34 to 112.15)	-0.89 (-1.07 to -0.72)
Germany	54,565 (51,824 to 57,576)	38,544 (34,664 to 42,955)	-29.36 (-37.27 to -20.71)	136.81 (129.94 to 144.36)	107.38 (96.57 to 119.67)	-0.65 (-1.37 to 0.08)
India	196,518 (173,031 to 222,724)	485,009 (404,897 to 574,274)	146.80 (95.58 to 198.98)	46.78 (41.19 to 53.02)	63.85 (53.30 to 75.60)	0.90 (0.78 to 1.02)
Indonesia	81,640 (59,585 to 99,878)	204,753 (146,965 to 268,911)	150.80 (89.18 to 238.01)	86.35 (63.02 to 105.64)	143.39 (102.92 to 188.32)	1.76 (1.56 to 1.97)
Italy	37,010 (36,008 to 38,025)	28,751 (26,959 to 30,462)	-22.32 (-27.02 to -17.69)	128.85 (125.36 to 132.39)	113.37 (106.31 to 120.12)	-0.55 (-0.62 to -0.47)
Japan	108,179 (105,866 to 110,401)	69,581 (65,208 to 72,617)	-35.68 (-39.04 to -33.09)	166.53 (162.97 to 169.95)	134.44 (125.99 to 140.31)	-0.92 (-1.12 to -0.73)
Mexico	21,035 (20,578 to 21,489)	66,065 (56,183 to 76,697)	214.07 (167.79 to 267.27)	49.48 (48.41 to 50.55)	99.66 (84.76 to 115.70)	2.62 (2.49 to 2.74)
Republic of Korea	26,609 (24,593 to 28,523)	27,871 (24,461 to 31,520)	4.74 (-9.29 to 21.57)	102.81 (95.02 to 110.21)	107.00 (93.91 to 121.01)	-0.30 (-0.63 to 0.04)
Russian Federation	91,490 (83,363 to 97,226)	109,286 (92,474 to 127,282)	19.45 (2.36 to 38.43)	123.21 (112.27 to 130.94)	159.61 (135.05 to 185.89)	0.08 (-0.41 to 0.59)
Saudi Arabia	3731 (2433 to 5575)	24,229 (17,410 to 33,729)	549.37 (272.92 to 1024.77)	46.15 (30.09 to 68.96)	99.47 (71.48 to 138.47)	2.61 (2.56 to 2.65)
South Africa	17,077 (15,159 to 19,343)	25,406 (21,885 to 29,487)	48.77 (21.35 to 85.16)	89.68 (79.61 to 101.58)	83.43 (71.87 to 96.83)	-0.59 (-0.85 to -0.34)
Turkey	36,606 (27,503 to 46,951)	50,299 (39,384 to 63,595)	37.41 (-4.11 to 96.89)	122.26 (91.85 to 156.81)	110.16 (86.25 to 139.28)	-0.79 (-1.21 to -0.36)
United Kingdom	37,344 (36,500 to 38,238)	34,838 (33,720 to 36,016)	-6.71 (-10.36 to -3.33)	131.13 (128.17 to 134.27)	114.29 (110.63 to 118.16)	-0.50 (-0.66 to -0.34)
United States of America	160,880 (156,448 to 165,077)	205,248 (197,214 to 213,730)	27.58 (21.95 to 35.16)	120.19 (116.88 to 123.33)	136.33 (130.99 to 141.96)	0.48 (0.33 to 0.62)

*G20* Group of 20, *UI* Uncertainty interval, *EAPC* Estimated annual percentage change, *CI* Confidence interval, *DALY* Disability-adjusted life years

### Temporal trends in EOCRC burden over time

With the exception of the Germany and South Africa, the ASIRs for EOCRC in all the other G20 member countries increased during 1990–2019. In China, the ASIR for EOCRC presented an increasing trend with an EAPC being 4.61 (95% *CI*: 4.45–4.77) (Table 1). The ASPRs increased in all G20 countries, being highest in Saudi Arabia (EAPC = 5.90, 95% *CI*: 5.67–6.14), followed by China (EAPC = 5.82, 95% *CI*: 5.60–6.05) and Mexico (EAPC = 4.04, 95% *CI*: 3.91–4.17) (Table 2). In terms of ASMRs for EOCRC during 1990–2019, China, Argentina, Brazil, India, Indonesia, Mexico, Saudi Arabia, and United States of America had upward trends, whereas the descending trends were observed in Australia, European Union, France, Italy, Japan, South Africa, Turkey, and United Kingdom. Meanwhile, the ASMRs in Canada, Germany, Republic of Korea, and Russian Federation remained stable (Table 3). The age-standardized DALY rate of EOCRC in China increased (EAPC = 1.64, 95% *CI*: 1.54–1.75), moreover, China’s upward trend ranked 4^th^ in the G20 countries, followed by Mexico, Saudi Arabia, and Indonesia (Table 4).

### Risk factors for DALYs of EOCRC in China

**Fig. 1:**
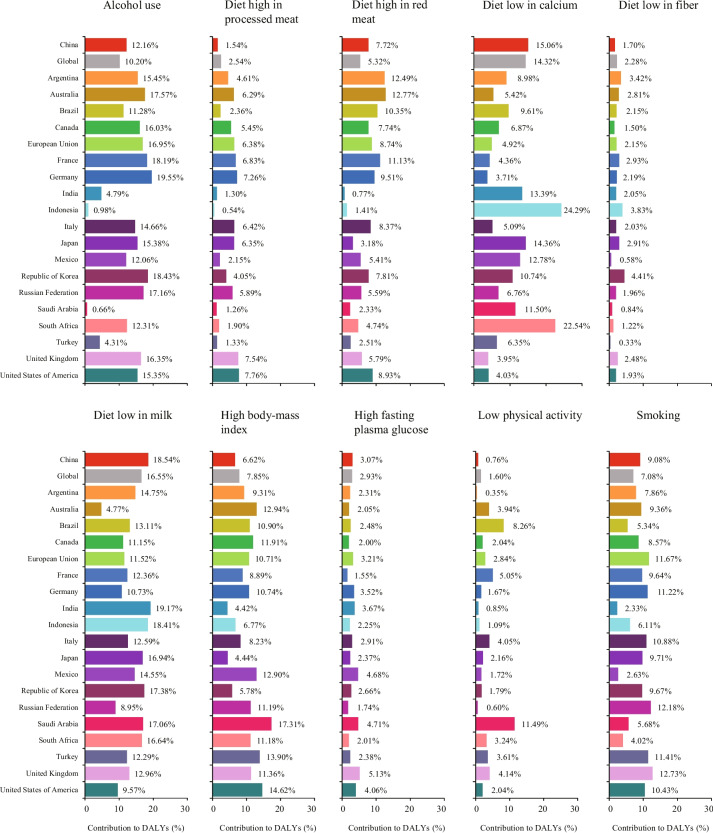
The proportion of DALYs due to EOCRC attributable to risk factors in China and other G20 countries in 2019. DALYs: Disability-adjusted life years; EOCRC: Early-onset colorectal cancer; G20: Group of 20

Although the DALYs of EOCRC in China was attributed to different individual risk factors, the five major risk factors in 2019 were diet low in milk [18.54% (95% UI: 12.71–24.07)], diet low in calcium [15.06% (95% UI: 10.70–20.03)], alcohol use [12.16% (95% UI: 8.87–15.64)], smoking [9.08% (95% UI: 3.39–14.11)], and diet high in red meat [9.08% (95% UI: 3.39–14.11)] (Fig. 1). The proportions of DALYs attributable to risk factors differed by sex and country. The five most common risk factors for males were diet low in milk, alcohol use, diet low in calcium, smoking, and high body-mass index, whereas diet low in milk, diet low in calcium, diet high in red meat, high body-mass index, and high fasting plasma glucose were the five risk factors that contributed most to DALYs in females (Figs. S1 and S2). At the national level, the highest percentage of DALYs for alcohol use was in Germany [19.55% (95% UI: 14.93–24.34)], for diet high in processed meat was in United States of America [7.76% (95% UI: 2.90–13.38)], for diet high in red meat was in Australia [12.77% (95% UI: 6.34–18.92)], for diet low in calcium was in Indonesia [24.29% (95% UI: 19.73–29.01)], for diet low in fiber was in Republic of Korea [4.41% (95% UI: 1.74–7.36)], for diet low in milk was in India [19.17% (95% UI: 13.68–24.61)], for high body-mass index was in Saudi Arabia [17.31% (95% UI: 10.89–23.84)], for high fasting plasma glucose was in United Kingdom [5.13% (95% UI: 1.14–11.50)], for low physical activity was in Saudi Arabia [11.49% (95% UI: 3.71–19.37)], and for smoking was in United Kingdom [12.73% (95% UI: 4.65–19.60)] (Fig. 1).

### Predictions of incidence, mortality and DALYs of EOCRC in China from 2020 to 2029

**Fig. 2:**
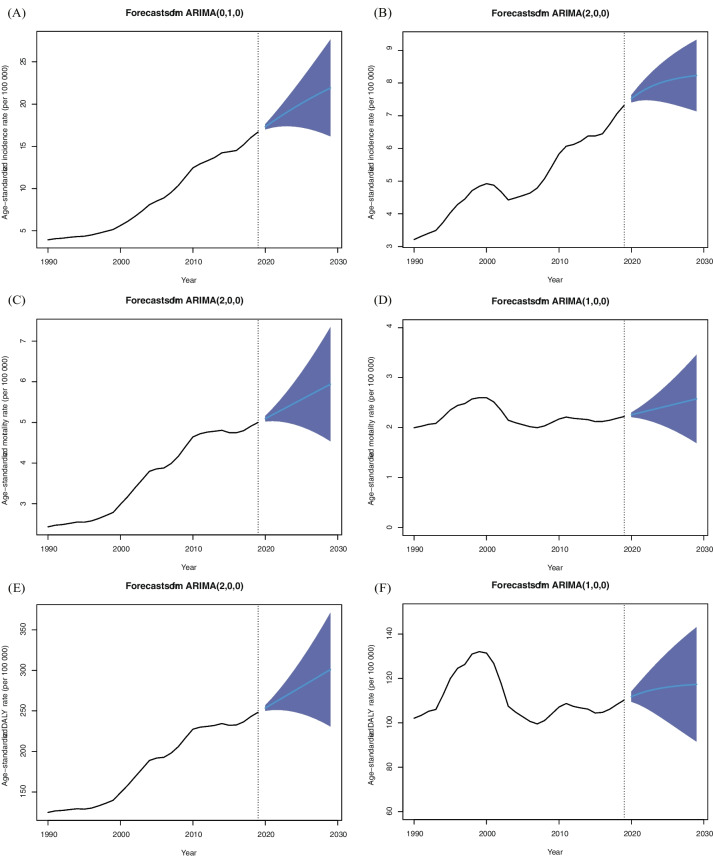
The temporal trends in the age-standardized rates for incidence, mortality, and DALY of EOCRC from 1990 to 2019 and projections from 2020 to 2029 in China, stratified by gender. **A** The temporal trends in the age-standardized incidence rate of EOCRC from 1990 to 2019 and projections from 2020 to 2029 in males. **B** The temporal trends in the age-standardized incidence rate of EOCRC from 1990 to 2019 and projections from 2020 to 2029 in females. **C** The temporal trends in the age-standardized mortality rate of EOCRC from 1990 to 2019 and projections from 2020 to 2029 in males. **D** The temporal trends in the age-standardized mortality rate of EOCRC from 1990 to 2019 and projections from 2020 to 2029 in females. **E** The temporal trends in the age-standardized DALY rate of EOCRC from 1990 to 2019 and projections from 2020 to 2029 in males. **F** The temporal trends in the age-standardized DALY rate of EOCRC from 1990 to 2019 and projections from 2020 to 2029 in females. DALYs: Disability-adjusted life years; EOCRC: Early-onset colorectal cancer

We used the ARIMA model to fit the ASIR, ASMR, and age-standardized DALY rate of EOCRC stratified by sex, from 1990 to 2019 and predict them in 2029. The selected optimal model parameters and corresponding AIC and BIC were presented in the Table S1. By 2029, the ASIR in males will increase to 21.91 (95% *CI*: 16.18–27.64) per 100,000 population with an EAPC of 2.63 (95% *CI*: 2.47–2.79) (Table 5, Fig. 2A). The ASIR in females will reach 8.23 (95% *CI*: 7.14–9.32) per 100,000 population, which means a slight increase with an EAPC of 0.95 (95% *CI*: 0.76–1.15) (Table 5, Fig. 2B). Men are anticipated to have a steeper increase than women in the ASMR during 2020–2029, with EAPCs of 1.73 (95% *CI*: 1.70–1.76) and 1.45 (95% *CI*: 1.42–1.49), respectively (Table 5, Fig. 2C, D). Meanwhile, the increasing speed of rise in the age-standardized DALY rate in males with an EAPC of 1.92 (95% *CI*: 1.89–1.96) will be higher than in females with an EAPC of 0.51 (95% *CI*: 0.41–0.61) (Table 5, Fig. 2E, F).

**Table 5:** Predictions of incidence, mortality, and DALYs of early-onset colorectal cancer in China stratified by sex, from 2020 to 2029

**Year**	**Age-standardized incidence rate, per 100 000 (95% CI)**		**Age-standardized mortality rate, per 100 000 (95% CI)**		**Age-standardized DALY rate, per 100 000 (95% CI)**	
	**Male**	**Female**	**Male**	**Female**	**Male**	**Female**
2020	17.32 (17.01 to 17.63)	7.52 (7.41 to 7.64)	5.09 (5.02 to 5.16)	2.26 (2.21 to 2.30)	253.58 (250.17 to 256.99)	111.82 (109.49 to 114.15)
2021	17.92 (17.23 to 18.60)	7.69 (7.46 to 7.91)	5.19 (5.03 to 5.34)	2.29 (2.19 to 2.39)	258.85 (251.19 to 266.51)	113.08 (108.27 to 117.89)
2022	18.49 (17.36 to 19.63)	7.82 (7.48 to 8.15)	5.28 (5.02 to 5.54)	2.33 (2.16 to 2.50)	264.13 (251.25 to 277.00)	114.10 (106.61 to 121.58)
2023	19.05 (17.39 to 20.70)	7.92 (7.47 to 8.38)	5.38 (5.00 to 5.76)	2.36 (2.11 to 2.61)	269.40 (250.45 to 288.35)	114.91 (104.68 to 125.13)
2024	19.58 (17.35 to 21.80)	8.01 (7.44 to 8.57)	5.47 (4.95 to 5.99)	2.40 (2.06 to 2.73)	274.67 (248.88 to 300.47)	115.56 (102.59 to 128.52)
2025	20.08 (17.24 to 22.93)	8.07 (7.39 to 8.75)	5.56 (4.90 to 6.23)	2.43 (2.00 to 2.86)	279.95 (246.58 to 313.31)	116.07 (100.40 to 131.75)
2026	20.57 (17.06 to 24.08)	8.13 (7.34 to 8.92)	5.66 (4.83 to 6.49)	2.47 (1.93 to 3.00)	285.22 (243.61 to 326.83)	116.49 (98.17 to 134.81)
2027	21.04 (16.82 to 25.25)	8.17 (7.27 to 9.06)	5.75 (4.74 to 6.76)	2.50 (1.86 to 3.15)	290.49 (239.97 to 341.01)	116.82 (95.93 to 137.71)
2028	21.49 (16.53 to 26.44)	8.20 (7.21 to 9.20)	5.85 (4.65 to 7.05)	2.54 (1.78 to 3.30)	295.77 (235.70 to 355.83)	117.09 (93.70 to 140.47)
2029	21.91 (16.18 to 27.64)	8.23 (7.14 to 9.32)	5.94 (4.54 to 7.34)	2.57 (1.69 to 3.46)	301.04 (230.81 to 371.27)	117.30 (91.50 to 143.10)
2020–2029 EAPC (95% CI)	2.63 (2.47 to 2.79)	0.95 (0.76 to 1.15)	1.73 (1.70 to 1.76)	1.45 (1.42 to 1.49)	1.92 (1.89 to 1.96)	0.51 (0.41 to 0.61)

*EAPC* Estimated annual percentage change, *CI* Confidence interval, *DALY* Disability-adjusted life years

## Discussion

The present study provided the up-to-date estimates on incidence, prevalence, mortality, DALYs, and leading risk factors associated with EOCRC in China and compared these rates to other G20 countries over a 30-year period during 1990–2019, while utilizing data collected from the GBD 2019. The G20 is an international economic cooperation forum composed of 20 developed and developing countries. Similar to people in other countries around the world, the burden of diseases in G20 countries is inseparable from their specific economic circumstances and health conditions [27, 28]. It is estimated that 71% of the world’s elderly live in G20 countries, and most G20 member countries have experienced the high level of mortality attributed to non-communicable diseases including EOCRC. Thus, G20 countries should play a crucial role in helping to improve global health due to member countries have been confronted with many issues associated with aging society and increased burden of non-communicable diseases.

We observed that the increase in ASIR and ASPR over the past 30 years occurred in most G20 countries, which was consistent with previous studies [29–31]. In China, the incidence, prevalence, mortality, and DALYs of EOCRC have been on the rise from 1990 to 2019. Indeed, the estimated number of incident and prevalent EOCRC cases was 87,383 and 590,804 in 2019, which accounted for 38.71% and 41.57% of all EOCRC cases globally. Compared with the global average levels and other members of the G20 countries, the ASIR and ASPR of EOCRC increased significantly with EAPCs of 4.61 (95% *CI*: 4.45 to 4.77) and 5.82 (95% *CI*: 5.60 to 6.05) in China during the study period. The reasons underlying this rise of EOCRC are unclear. One possible hypothesis is that early-life exposure to established CRC risk factors including westernized diet, high body-mass index, and physical inactivity might result in genetic and epigenetic changes in colorectal epithelial cells, gut microbiota, and host immunity [10]. Although the role of genetic factors in the pathogenesis of EOCRC is more evident than that of LOCRC, most EOCRC patients are still sporadic [32]. Our findings indicated that proportions of EOCRC DALYs attributable to dietary, behavioral, and metabolic risk factors in China were 76.25% in 2019 (Fig. 1), while attributable risk proportions of dietary, behavioral, and metabolic factors in males were higher than those in females (86.27% *vs*. 52.58%) (Figs. S1 & S2). Therefore, the increase in incidence rate is mainly due to the increased exposure of risk factors. We further found that diet low in milk and diet low in calcium were the top two risk factors for EOCRC in 2019. High dietary milk and calcium intakes were reported to be associated with the reduced risk of CRC and EOCRC, possibly due to vitamin D and calcium may activate signaling pathways that are involved in regulating the inhibition of epithelial cell proliferation, induction of target tissue differentiation, regulation of antioxidant enzyme gene expression, and induction of carcinoma cell apoptosis via calcium-sensing receptor through promoting of E-cadherin expression, suppressing of β-catenin/T cell factor activation, and activating of p38 mitogen-activated protein kinase cascade [33–35]. Alcohol use and smoking contributed considerably to the burden of EOCRC. Many epidemiologic investigations have identified that alcohol use and smoking are associated with increased risks of EOCRC in a dose-dependent manner [36–38]. Ethanol and related metabolites initiate multiple signaling cascades augmenting the cancer progression, such as DNA-adduct formation, oxidative stress and lipid peroxidation, epigenetic alterations, epithelial barrier dysfunction, and immune modulatory effects [39]. Moreover, smoking promotes colon carcinogenesis possibly through impairing the phagocytic function of macrophages and augmenting the function of M2-like macrophages, which were associated with worse cancer-specific survival of CRC [40, 41]. A positive association between diet high in red meat and alkylating signatures was found in the distal colorectum. These alkylating signatures targeted cancer driver mutations *KRAS* p.G12D, *KRAS* p.G13D, and *PIK3CA* p.E545K, as well as predicted poor survival in CRC patients [42]. Additionally, changes in lifestyle and dietary habits such as high sugar-sweetened beverage consumption in adolescence and young adulthood are also associated with increased risk of EOCRC [10, 43].

From 1990 to 2019, the trends of age-standardized DALY rates for EOCRC showed patterns similar to those of ASMRs in the G20 countries. In contrast to the increasing trends of ASIR and ASPR in most G20 countries, ASMR and age-standardized DALY rate due to EOCRC declined or increased at a relatively low pace. This was consistent with a recent study, which revealed that mortality rates of EOCRC showed a downward trend in the high SDI countries, particularly in Australia, France, Germany, Japan, and the United Kingdom [19]. The amelioration in ASMR is possibly attributed to the continuous progression of early screening and efficient treatment approaches for CRC, coupled with the popularization of multidisciplinary comprehensive treatment concepts, including surgery, radiotherapy, chemotherapy, targeted therapy, and immunotherapy [44]. Although EOCRC patients were associated with a higher risk of malignancy and late staging compared to the LOCRC, EOCRC patients had fewer complications and higher tolerance and acceptance of cancer therapy, which was also beneficial for improving the treatment effectiveness and survival [45]. In most countries, early screening for CRC is currently performed in individuals over 50 years old. However, due to the rising incidence of EOCRC, some expert panels such as US Preventive Services Task Force recommended to lower the screening initiation age to 45 years [46, 47]. China has initiated some population-based CRC screening programs, such as a large-scale screening for high-risk populations in Haining County in the 1970s and the Cancer Screening Program in Urban China (CanSPUC) in 2012, which effectively inhibit the substantial increase of mortality of CRC in China [48]. However, according to the forecast results of ARIMA model, the ASIR, ASMR, and age-standardized DALY rate of EOCRC will still increase over the next 10 years in China. Moreover, men will experience higher ASIR, ASMR, and age-standardized DALY rate and have more significantly increasing trends of the rates compared with women. Thus, promising approaches to optimize prevention and early detection of EOCRC including the screening of high-risk populations such as young adults with family history of CRC in first-degree relatives should be emphasized.

Several limitations should be acknowledged in the present study. First, the data provided in GBD 2019 were based on estimation and mathematical modelling, which may affect the accuracy and reliability of the burden estimates. Second, due to detailed data about burden and trends of EOCRC stratified by histological subtype were not available, we were unable to assess the burden of colon cancer and rectal cancer respectively. Third, some potential risk factors of EOCRC such as antibiotic usage and changes in the gut microbiome were not analyzed due to the limitations of relevant data sources. Fourth, the absence of provincial-level data regarding the burden of EOCRC may not reflect the regional and provincial disparities of disease burden in China.

## Conclusion

The present study carried out a comprehensive and in-depth analysis regarding the burden and long-term trend of EOCRC in China and other G20 countries. The ASIR, ASPR, ASMR, and age-standardized DALY rate of EOCRC increased from 1990 to 2019 in China. There were substantial differences in these rates among G20 countries. Diet low in milk, diet low in calcium, alcohol use, smoking, and diet high in red meat were the five leading risk factors for DALYs of EOCRC in 2019, which should be paid more attention to. The burden of EOCRC is predicted to increase continuously over the next 10 years in China. The huge burden of EOCRC calls for close collaboration and cooperation amongst G20 countries to implement interventions for improving early detection, optimizing early-stage diagnosis, and developing effective treatment and prevention strategies to reduce the burden of EOCRC.

## Supplementary Information

**Additional file 1: Figure S1.** The proportion of DALYs due to EOCRC attributable to risk factors in China and other G20 countries in males in 2019. DALYs: Disability-adjusted life years; EOCRC: Early-onset colorectal cancer; G20: Group of 20.

**Additional file 2: Figure S2.** The proportion of DALYs due to EOCRC attributable to risk factors in China and other G20 countries in females in 2019. DALYs: Disability-adjusted life years; EOCRC: Early-onset colorectal cancer; G20: Group of 20.

**Additional file 3: Table S1.** ARIMA model parameters and their corresponding AIC and BIC for prediction of age-standardized rate (per 100 000) of all three measures for early-onset colorectal cancer for the next 10 years in China.

## Data Availability

Publicly available datasets were analyzed in this study. The data can be found here: http://ghdx.healthdata.org/gbd-results-tool.
