# Supplementary figures and images for: Burden of early-onset colorectal cancer along with attributable risk factors from 1990 to 2019: a comparative study between China and other G20 countries
Source: BMC Public Health. 2023 Jul 31;23:1463. doi: 10.1186/s12889-023-16407-y (PMC10391986; doi:10.1186/s12889-023-16407-y)

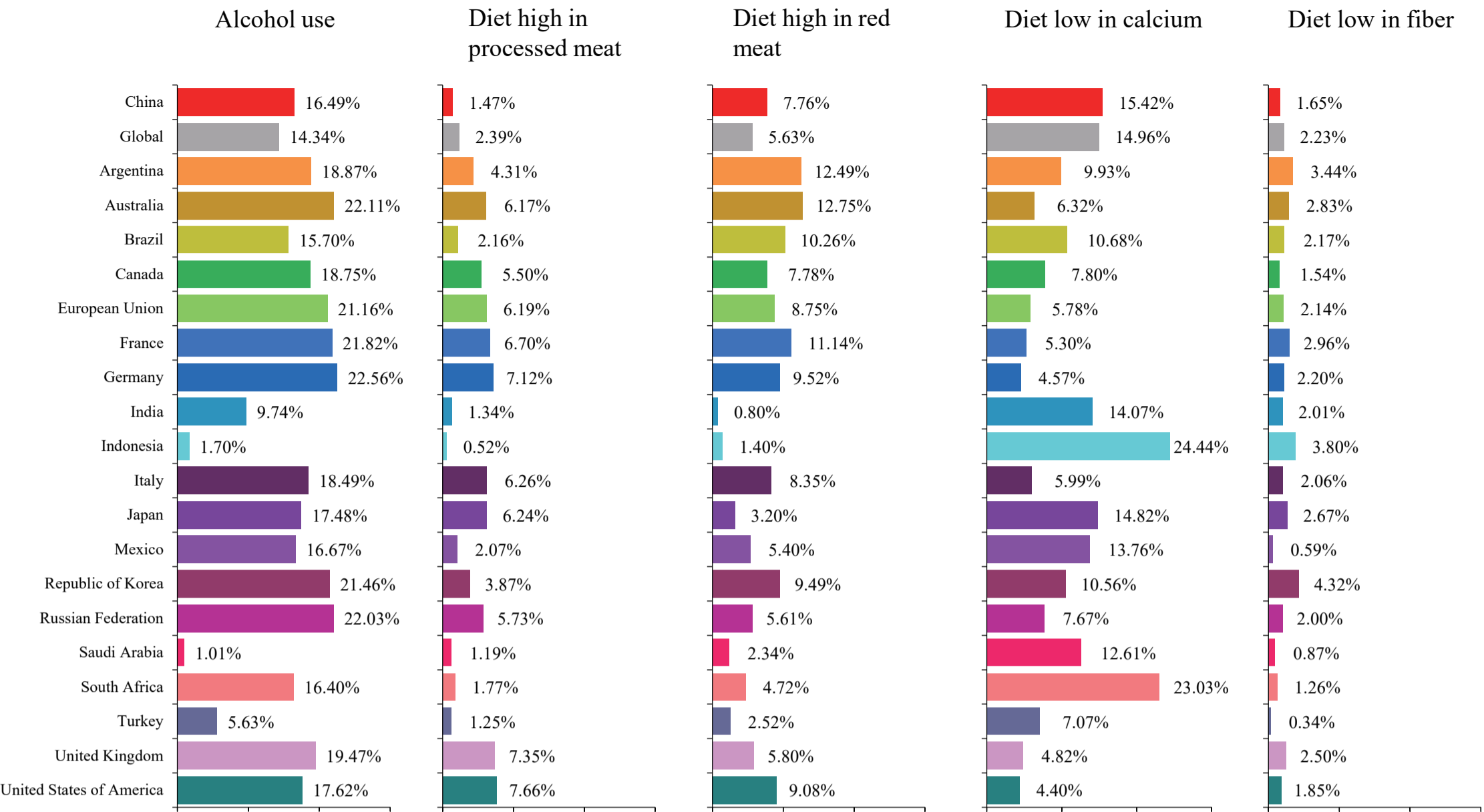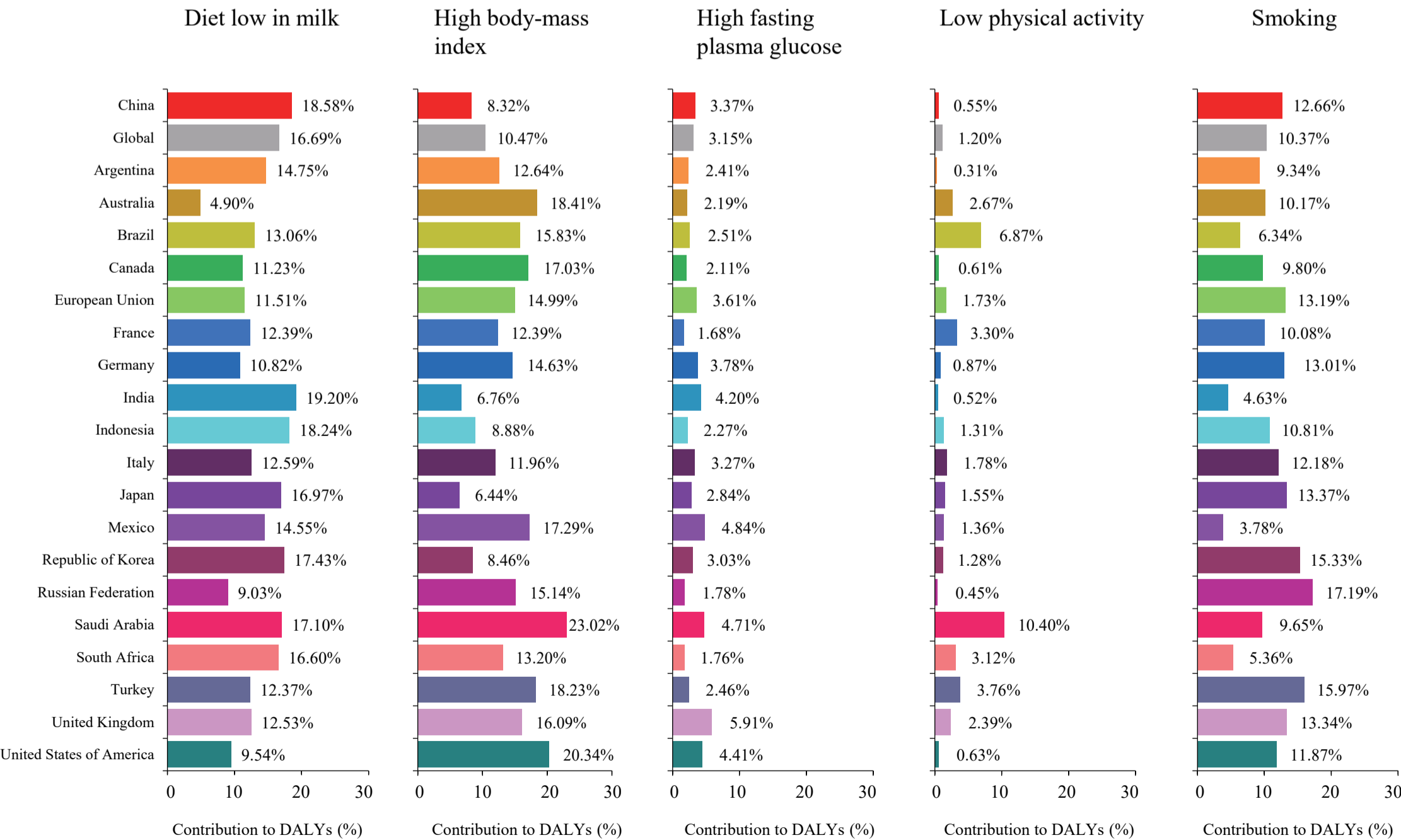

Supplement: Supplementary file 1 — Additional file 1: Figure S1. The proportion of DALYs due to EOCRC attributable to risk factors in China and other G20 countries in males in 2019. DALYs: Disability-adjusted life years; EOCRC: Early-onset colorectal cancer; G20: Group of 20. [file 12889_2023_16407_MOESM1_ESM.pdf]

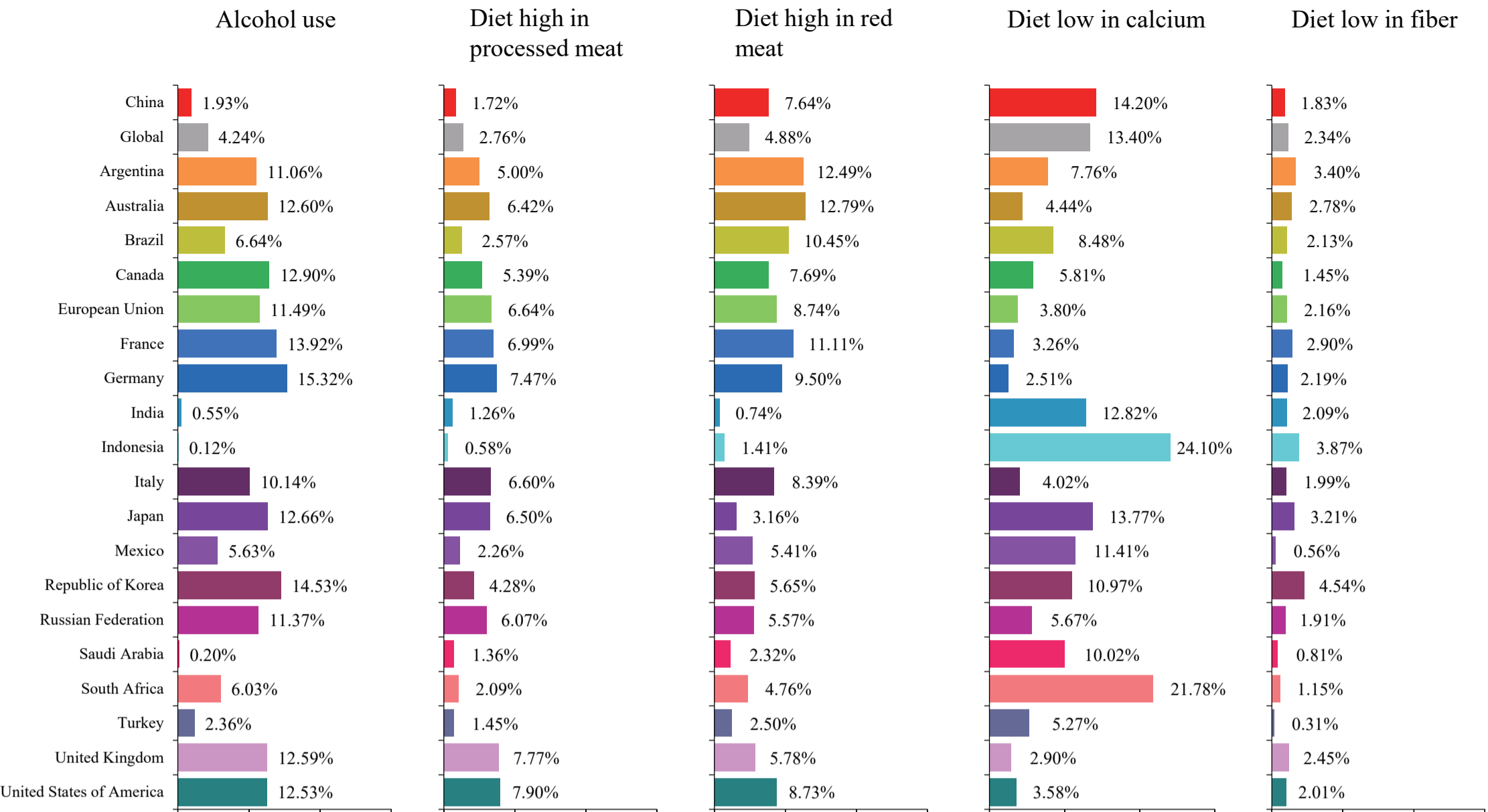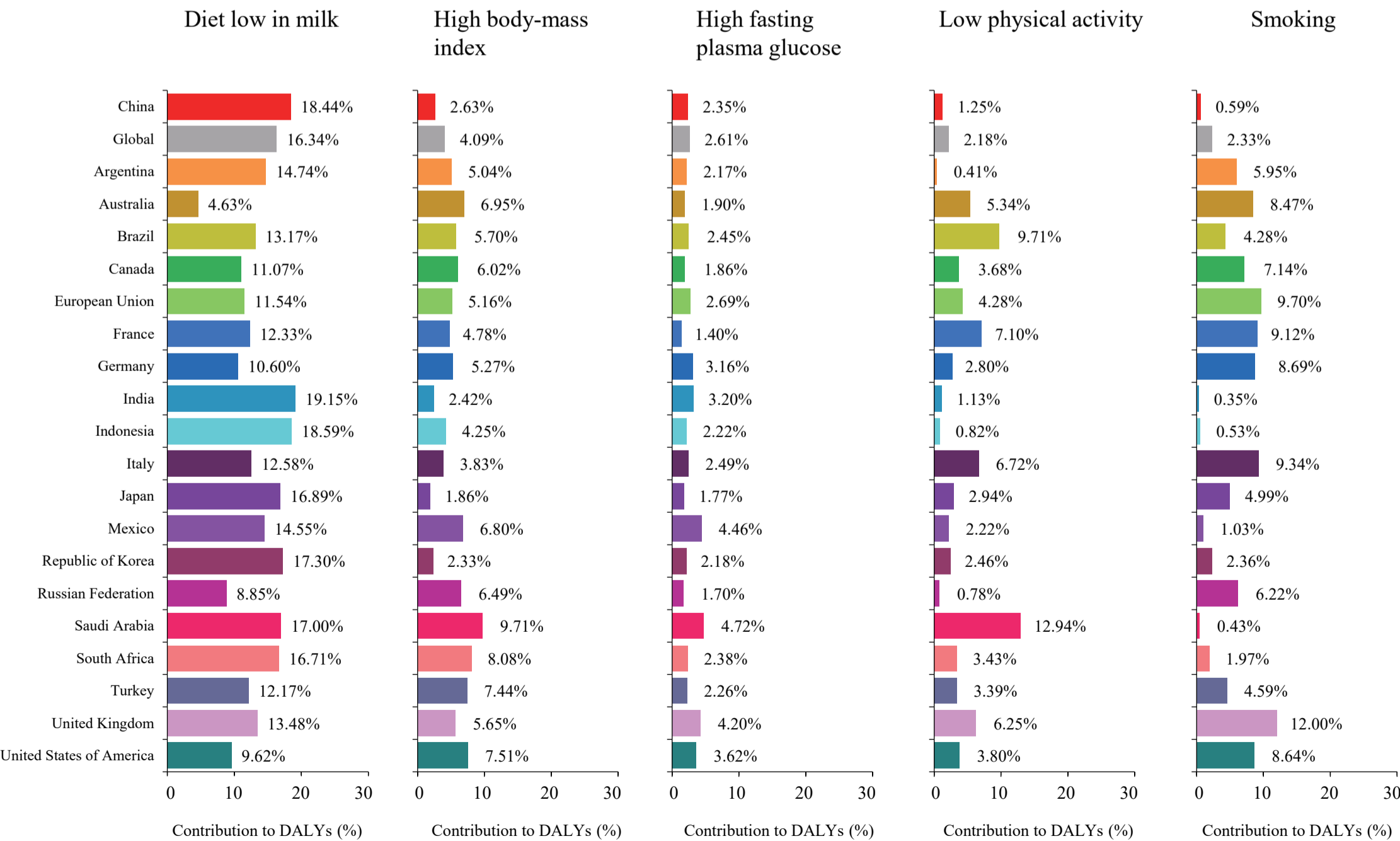

Supplement: Supplementary file 2 — Additional file 2: Figure S2. The proportion of DALYs due to EOCRC attributable to risk factors in China and other G20 countries in females in 2019. DALYs: Disability-adjusted life years; EOCRC: Early-onset colorectal cancer; G20: Group of 20. [file 12889_2023_16407_MOESM2_ESM.pdf]
