# Supplementary material for: Burden of early-onset colorectal cancer along with attributable risk factors from 1990 to 2019: a comparative study between China and other G20 countries
Source: BMC Public Health. 2023 Jul 31;23:1463. doi: 10.1186/s12889-023-16407-y (PMC10391986; doi:10.1186/s12889-023-16407-y)
Supplement: Supplementary file 3 — Additional file 3: Table S1. ARIMA model parameters and their corresponding AIC and BIC for prediction of age-standardized rate (per 100 000) of all three measures for early-onset colorectal cancer for the next 10 years in China. [file 12889_2023_16407_MOESM3_ESM.docx]

**Additional file 3: Table S1** ARIMA model parameters and their corresponding AIC and BIC for prediction of age-standardized rate (per 100 000) of all three measures for early-onset colorectal cancer for the next 10 years in China

| **Sex** | **Age-standardized incidence rate** | | |  | **Age-standardized mortality rate** | | |  | **Age-standardized DALY rate** | | |
| --- | --- | --- | --- | --- | --- | --- | --- | --- | --- | --- | --- |
|  | Parameters | AIC | BIC |  | Parameters | AIC | BIC |  | Parameters | AIC | BIC |
| Male | ARIMA (0,1,0) | 33.16 | 35.01 |  | ARIMA (2,0,0) | 1.09 | 5.21 |  | ARIMA (2,0,0) | -14.08 | -13.15 |
| Female | ARIMA (2,0,0) | 24.09 | 25.41 |  | ARIMA (1,0,0) | 4.43 | 1.71 |  | ARIMA (1,0,0) | -26.92 | -25.89 |

*AIC* Akaike information criterion, *BIC* Bayesian information criterion, *DALY* Disability-adjusted life year
